# Vasotocin receptor gene genotypes moderate the relationship between cortical thickness and sensory processing

**DOI:** 10.1038/s41398-023-02657-2

**Published:** 2023-11-21

**Authors:** Seonkyoung Lee, Yongjeon Cheong, Yeseul Ryu, Hirotaka Kosaka, Minyoung Jung

**Affiliations:** 1 Cognitive Science Research Group, Korea Brain Research Institute, Daegu, Republic of Korea; 2 Department of Neuropsychiatry, University of Fukui, Eiheiji, Fukui Japan; 3 Division of Developmental Higher Brain Functions, United Graduate School of Child Development, University of Fukui, Eiheiji, Japan

**Keywords:** Diagnostic markers, Human behaviour

## Abstract

Sensory processing is the process by which the central nervous system gathers, interprets, and regulates sensory stimuli in response to environmental cues. However, our understanding of the genetic factors and neuroanatomical correlations that influence sensory processing is limited. The vasotocin system modulates sensory input responsiveness, making it a potential candidate for further investigation. Additionally, human neuroimaging studies have demonstrated that the ability to modulate sensory stimuli is related to neuroanatomical features such as cortical thickness. Therefore, this study aimed to examine the relationship between functional polymorphisms in vasotocin receptor (VTR) genes, sensory profiles, and neuroanatomical correlations. We used structural magnetic resonance imaging (MRI) and the Adolescent/Adult Sensory Profile (AASP) questionnaire in 98 healthy adult participants to assess sensory processing and identified seven single nucleotide polymorphisms. We found that A-allele carriers of rs1042615 in VTR had higher scores for “sensory sensitivity” and “sensation avoiding”. Moreover, higher scores for three AASP subscales were associated with decreased cortical thickness in various regions, including the right precentral, paracentral, and fusiform gyri, as well as bilateral inferior temporal gyri. This study sheds light on the potential role of genetic variations in the VTR in modulating sensory processing and correlation with cortical thickness which has future implications for better understanding sensory abnormalities in neurodevelopmental disorders.

## Introduction

Sensory processing involves the accumulation, interpretation, and modulation of sensory information by the central nervous system [1, 2]. The brain processes and organizes extrinsic and intrinsic sensory information to appropriately respond to specific situational demands. Sensory input regulation requires a balance between excitation and inhibition (E/I balance). Moreover, a neurological threshold is important for adequately regulating sensory input [3]. All animals, including humans, have different sensory thresholds which lead to different behavioral patterns.

Considering sensory thresholds and human behavior, Dunn et al. developed a sensory processing model, which states that two levels of neurological thresholds for sensory inputs (i.e., high or low) interact with two behavioral strategies (i.e., passive or active) [3, 4]. Thus, four sensory-processing patterns exist in this model: “low registration”, “sensation seeking”, “sensory sensitivity”, and “sensation avoiding”.

Individuals with “low registration”, have higher neurological thresholds for sensory input and passive behavioral strategies and are characterized by reduced responsiveness to everyday sensory events [4]. In contrast, sensation seekers have a higher threshold and exhibit active behavioral strategies. These individuals actively seek intense stimuli to enhance their sensory richness [4]. In contrast, individuals with “sensory sensitivity” or “sensation avoiding” have a low neurological threshold for sensory stimuli (i.e., hypersensitivity) and experience discomfort with sensations. However, in the behavioral strategy dimension, individuals with “sensory sensitivity”, who adopt passive strategies, are less likely to show avoidance responses to sensory stimuli. Whereas sensory avoiders, who take on active strategies, attempt to limit their exposure to sensations. Based on Dunn’s sensory processing model, the Adolescent/Adult Sensory Profile (AASP) questionnaire was developed which reflects the quadrant dimensions of human behavioral responses to various sensory inputs encountered in everyday life [4]. Thus, the questionnaire has been used to assess individual differences in sensory profiles.

Nevertheless, our knowledge of sensory processing and brain mechanisms underlying these individual differences is limited. What drives individual differences in sensory processing? Exploring the relationship between human sensory behavior and neural mechanisms that underlie sensory processing may contribute to a better understanding of individual differences in sensory processing.

Recently, researchers have attempted to connect sensory profile characteristics with neuroanatomical features in healthy individuals [2, 5]. Specifically, a higher “sensory sensitivity” score was associated with an increase in gray matter volume in the left inferior and middle frontal gyri [2], whereas a “sensation seeking” score was positively correlated with gray matter volume in the parahippocampal cortex, precentral gyrus (PreCG) and inferior temporal gyrus (ITG), and cuneus regions, and cortical thickness of inferior frontal and postcentral gyri [5]. Similarly, the relationship between the AASP scores and the white matter microstructure of the caudate and the structure of the caudate nucleus have been investigated using diffusion tensor imaging [6]. This study found significant associations among axonal diffusivity (AD), mean diffusivity (MD), tactile sensation, and tactile sensory avoidance which suggests a relationship between neuroanatomical structure and sensory processing.

Studies using the pre-pulse inhibition (PPI) paradigm, a more direct method of examining neurological threshold levels compared to self-report questionnaires such as AASP, reported that the ability to modulate repetitive sensory stimuli is associated with increased cortical thickness, particularly in the primary somatosensory cortex [7–10]. PPI is based on the theory of sensory gating, which inhibits the processing of redundant or unrelated stimuli, directs processing resources to goal-related stimuli, and protects the organism [11, 12]. Moreover, sensory gating deficits and the inability to filter out repetitive and irrelevant information may reflect hypersensitivity to sensory stimuli [13] and are observed in various neuropsychiatric disorders, such as autism spectrum disorder (ASD) and schizophrenia [9, 14]. Human neuroimaging studies have shown that thicker the cortex, the better it suppresses redundant stimuli [7]. In addition, patients with schizophrenia have a thinner auditory cortex [9] and reduced hippocampal volume [14], which suggests poor auditory sensory gating.

Meanwhile, studies investigating sensory gating using the PPI paradigm have revealed the involvement of genes [15, 16]. More specifically, one study examined the connection between vasotocin receptor 1A (also known as arginine vasopressin receptor 1 A) and sensory processing in 113 nonclinical adults. They found that a longer RS3 allele was associated with better sensory gating, highlighting the potential role of genes in modulating sensory processing [15]. In contrast, vasotocin receptor 1B knockout male mice displayed a deficit in filtering sensory and motor information [16]. Additionally, alterations in vasotocin or its receptors may indicate social behavioral deficits with sensory abnormalities (e.g., ASD and schizophrenia) [17, 18].

Vasotocin (VT) or arginine vasopressin (AVP) is a peptide hormone secreted by the posterior pituitary gland that binds to one of the three receptors: V1A, V1B, or V2, with V1 receptors located in the brain [17, 19]. Hereafter the term vasotocin will be used following the recent nomenclature recommended by Theofanopoulou et al. [19]. In addition to its role as a neuropituitary hormone, VT functions as a neuropeptide that can produce behavioral effects when released within a specific circuit in the brain [20–23]. While VT is known to contribute to various social behaviors [15–18, 20–33], recent evidence highlights its involvement in sensory modulation to elicit appropriate social behaviors [15, 17, 23, 33, 34]. Sensory processing and modulation by VT are linked to larger regulatory networks and execute contextually appropriate behavior [23]. Recently, Mulholland et al. suggested that VTR1A polymorphisms are associated with structural variations within the primate social brain network [35]. However, the role of the vasotocin receptor (VTR) in modulating and interpreting sensory information has been primarily studied only in animal models [17, 23, 34].

Previous studies have separately investigated the relationship between morphological features of the brain and sensory processing, as well as genes and sensory gating. However, it is currently unclear how these factors are interrelated in a unified framework. Given the role of VTR in the regulation of sensory stimuli [15, 17, 33], it is important to investigate the involvement of genetic factors in sensory processing and brain mechanisms together. This can provide valuable insights into the nature of individual differences in sensory processing. Based on this, the present study investigated the polymorphism of the VTR gene and its association with brain structure and sensory characteristics. Using the AASP, the present study aimed to examine the neuroanatomical correlations between sensory profiles and VTR gene polymorphisms in humans. Specifically, we hypothesized that the four sensory profile scores measured by the AASP differ depending on VTR gene polymorphisms and investigated the correlations between these scores and morphological features of the brain.

## Methods

### Participants

In total, 98 healthy adults (44 males, 54 females; mean age: 26.8 ± 6.8, 19–43 years) participated in this study. The patient exclusion criteria were as follows: 1) history of major physical or neurological illnesses, 2) history of head trauma, 3) history of problems with medication and drug abuse, 4) full-scale intelligence quotient scores below 80 (measured using the Wechsler Adult Intelligence Scale, 3^*rd*^ edition), and 5) left-handedness.

This study was approved by The Research Ethics Committee of University of Fukui (Assurance no. 20200081). The study was performed in accordance with the ethical standards of the Declaration of Helsinki. After explaining the purpose and procedure of the study, written informed consent was obtained from all participants.

### Adolescent/Adult Sensory Profiles (AASP)

The AASP self-report questionnaire was used to evaluate the sensory processing patterns of each participant. The questionnaire consists of 60 questions, measuring how individuals respond to sensory stimuli derived from everyday experiences on a 5-point Likert scale. Our study used the Japanese version of AASP that show high reliability and validity has been confirmed in Japanese adult cohorts (all dimension of Cronbach’s alpha >0.80) [36].

It has six subscales which assess response patterns in the taste/smell (8 items), visual (10 items), auditory (11 items), touch (13 items), movement (8 items), and activity level (10 items) domains. Notably, the items in each subscale are arranged to reflect the four sensory profiles (i.e., “low registration”, “sensation seeking”, “sensory sensitivity”, and “sensation avoiding”) in Dunn’s model. Each of the four quadrants relates to 15 statements and is calculated by summing the raw scores rated by participants. A higher total score signifies a more pronounced inclination towards atypical sensory processing, aligned with each quadrant [6, 37].

### Brain imaging procedure

MRI data were acquired using an 8-channel radio frequency head coil in a 3-T PET/MR GE scanner at the University of Fukui, Japan. The images were acquired with the following parameters: repetition time, 6.38 ms; echo time, 1.99 ms; flip angle, 11°; field of view, 256 mm; matrix, 256 × 256; number of slices, 172; voxel dimension, 1.0 × 1.0 × 1.0 mm^3^.

The obtained T1-weighted images were processed using FreeSurfer v5.1.0 (http://surfer.nmr.mgh.harvard.edu/), a validated open-access software package. This software automates several procedures and allows for quantitative assessment of brain anatomy, including measurements of subcortical volume and cortical morphology, with a level of accuracy comparable to that of manual methods. Imaging processing included motion correction, non-uniform intensity normalization, talairach transform computation, intensity normalization, skull strip, non-liner volumetric registration, and segmentation. The segmentation of the white, gray, and subcortical white matter was conducted in accordance with standard procedures using intensity, white-gray matter boundaries, and smoothness constraints [38]. Computation of cortical thickness, surface area, and brain volume measurement in 148 brain regions was based on a vertex model computational approach that involved dividing the cortical surface into the pial surface at each vertex of the cortex and spherical registration [39]. Based on previous studies [8, 40], we calculated individual cortical measurement (thickness, area, and volume) was calculated using average template surface with a spherical representation and fsaverage within the Destrieux Atlas template [41]. Sensory brain regions were selected by multiple comparisons at a false discovery rate (FDR) of *q* < 0.05 based on a total of 10 categories in the AASP. We performed a semi-automated quality control [42] using Qoala-T automatic detection [43] with visual inspection and editing as brain segmentation quality control.

### Genotyping

Saliva samples were obtained from all participants for DNA analysis. Genomic DNA was obtained from saliva using DNA self-collection kits OG-500 (DNA Genotek, Inc., Ottawa, Canada). VTR single-nucleotide polymorphism (SNP) was genotyped via real-time polymerase chain reaction (PCR) analysis using the StepOnePlus System (version 3.0.1.) and TaqMan genotyping with the assay-by-design method (Applied Biosystems, Foster City, CA, USA). Genotyping was performed in 10 µL volumes containing 9 ng genomic DNA, 0.25 µL of Tris-EDTA buffer, 0.25 µL of each TaqMan probe, and 5 µL TaqMan PCR Master Mix. The PCR cycling conditions comprised one 20 s cycle at 95°C, followed by 40 cycles at 95 °C for 3 s, and at 60 °C for 20 s. Finally, the following seven SNPs were selected for genotyping: rs1042615, rs3021528, rs10877969, and rs7268346 in VTR1A, and rs28632197, rs35630000, and rs33911258 in VTR1B (Table 1).

**Table 1:** Distributions by VTR SNPs genotype.

	SNP	Genotype	*N*	Genotype	*N*
VTR1A	rs1042615	AA/AG/GG	22/46/30	A/GG	68/30
	rs3021528	AA/AC/CC	72/24/2	AA/C	72/26
	rs10877969	CC/CT/TT	3/25/70	C/TT	28/70
	rs7268346	AA/AT/TT	3/25/70	A/TT	28/70
VTR1B	rs28632197	TT/TC/CC	18/80/0	TT/TC	18/80
	rs35630000	GG/GA/AA	77/20/1	GG/A	77/21
	rs33911258	AA/AG/GG	75/22/1	AA/G	75/23

*VTR* vasotocin receptor, *SNP* single-nucleotide polymorphism.

To address the limited sample size of the minor allele groups, we merged low-frequency homozygous and heterozygous individuals by following the analysis procedures in the previous studies regarding on effects of oxytocin receptor gene SNPs [44, 45]. For example, in the case of the rs33911258 SNP, only one participant exhibited the GG genotype, necessitating a combination of G-allele homozygotes and heterozygotes. This approach was utilized to enhance the statistical power and mitigate potential inference errors stemming from significant discrepancies in sample sizes among the genotype groups [44, 45].

### Statistical analysis

We performed a one-way analysis of covariance (ANCOVA) considering sex, age, and full-scale IQ as covariates to investigate potential VTR group differences in the sensory profiles. Subsequently, we performed a correlation analysis between the sensory profiles and three morphological features (i.e., surface area, cortical thickness, and volume) of the brain, depending on the genotype, to determine the effect of VTR on their association as follows. First, a partial correlation analysis was performed between the AASP scores and the three brain morphological features (FDR corrected of *q* < 0.05 based on a total of 10 categories in AASP) controlling for sex, age, and full-scale IQ. Second, Fisher’s r-to-z value transformation was performed to compare the differences in the correlation for each genotype. Statistical significance was set at *p* *≤* 0.005. All analyses were performed using SPSS version 21 and VassarStats software (http://vassarstats.net/).

## Results

### Behavioral results

Participant demographic data and the descriptive statistics for scores (mean, SD, range) across each sensory modality and quadrants by AASP are presented in Table 2.

**Table 2:** Demographic information.

	Mean	SD	Range
Subject (*N*)	98		
Age (years)	26.8	6.8	19–43
Sex (*N*, male/female)	44/54		
Full-scale IQ	112	11.6	83–135
Verbal IQ	111	12.1	77–139
Performance IQ	110	12.1	82–145
Adolescent/Adult Sensory Profile			
Quadrant scores			
Low registration	27.9	6.8	15–48
Sensation seeking	40.1	6.9	23–53
Sensory sensitivity	33.8	7.3	19–51
Sensation avoiding	33.6	7.5	17–49
Sensory modality-specific subscales			
Taste/smell	16.8	3.6	8–25
Movement	17.3	3.7	9–26
Visual	23.0	4.3	11–35
Touch	29.2	6.4	15–48
Activity level	26.2	4.9	14–37
Auditory	22.9	5.4	12–39

*SD* standard deviation, *IQ* intelligence quotient.

**Fig. 1:**
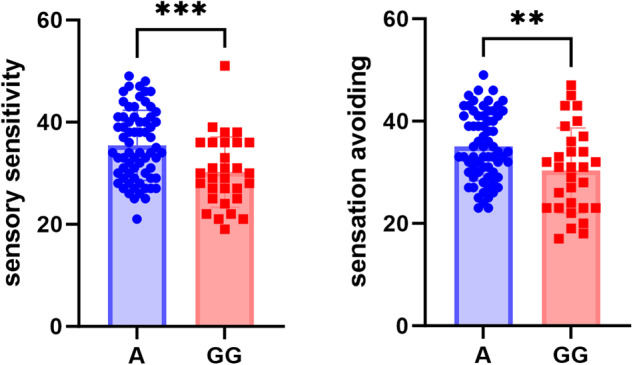
Bar graph of rs1042615 genotypic differences in two AASP scores. The A group scored higher on sensory sensitivity (left) and sensation avoiding (right) compared to the GG group (^***^p < 0.001, ^**^p < 0.01).

To ensure adequate statistical power, allele homozygotes with small sample sizes were merged with the heterozygous carriers [44, 45]. Among the seven genotyped SNPs, only one showed significant group differences in sensory characteristics. For rs1042615 A group reported higher scores than the GG group in the “sensory sensitivity’ (*F* (1, 93) = 11.273, *p* = 0.001) and “sensation avoiding’ (*F* (1, 93) = 8.028, *p* = 0.006) subscales (Fig. 1). Except for rs1042615, the other SNPs were not significant at the 0.05 significance level (Supplementary Table 1).

### Correlation analysis between brain morphological features and AASP scores

Given the notable distinctions observed in two sensory quadrants between rs1042615 A and GG groups, we performed partial correlation analyses to explore the relationship between sensory processing and the morphological feature (i.e., surface area, thickness, volume) in each group. No significant findings emerged concerning the cortical surface area and volume (Supplementary Table 2). However, distinctive relationships between AASP quadrant scores and cortical thickness were observed within the rs1042615 groups. Specifically, negative correlations between the three AASP subscales and cortical thickness were observed in the A-allele group but not in the GG group (Table 3). Additionally, negative correlations were found between cortical thickness in four regions (right paracentral [PCG], PreCG, fusiform gyri [FFG], and bilateral ITG) and three AASP scores (“low registration’, “sensory sensitivity”, and “sensation avoiding”).

**Table 3:** Pearson correlation coefficient (*r*) and *p* value between cortical thickness and AASP scores by SNP rs1042615 groups.

AASP	ROI (thickness)	A		GG		Group comparison
		*r*	*p*	*r*	*p*	*p*
Low registration	R.FFG	–0.421	0.000	0.273	0.160	0.002
	R.PreCG	–0.351	0.004	0.045	0.822	0.072
Sensory Sensitivity	R.FFG	–0.343	0.005	0.088	0.656	0.051
	R.PreCG	–0.382	0.002	0.047	0.812	0.050
	R.PCG	–0.400	0.001	0.320	0.097	0.001
	R.ITG	–0.343	0.005	–0.027	0.892	0.150
	L.ITG	–0.370	0.002	–0.120	0.544	0.242
Sensation avoiding	R.PreCG	–0.346	0.004	0.077	0.697	0.056
	R.PCG	–0.339	0.005	0.212	0.288	0.013

*R* right hemisphere, *L* left hemisphere, *FFG* right fusiform gyrus, *PreCG* precentral gyrus, *PCG* paracentral gyrus, *ITG* inferior temporal gyrus.

**Fig. 2:**
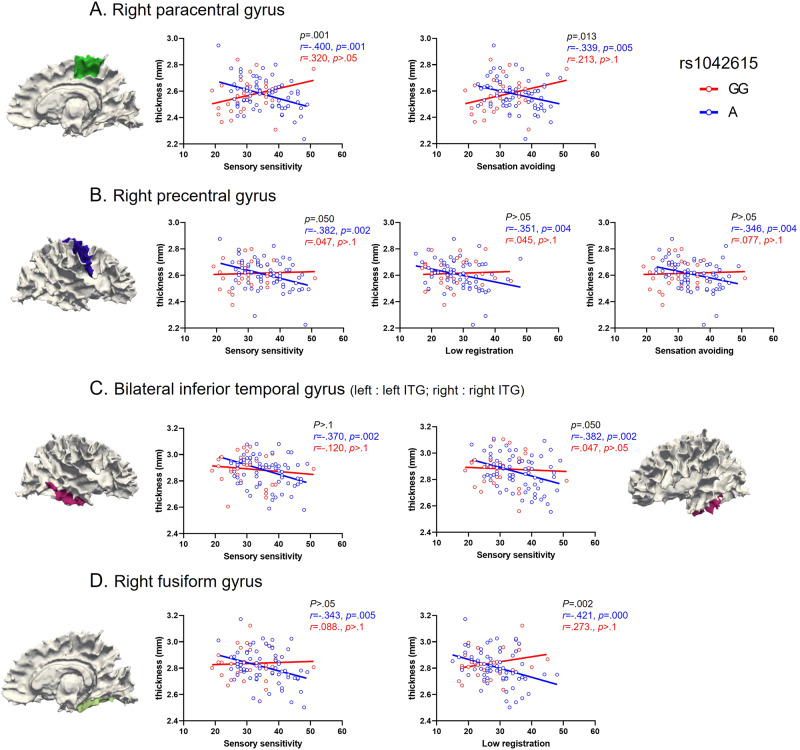
Relationship between cortical thickness and AASP scores in SNP rs1042615 groups. **A** Right paracentral gyrus, **B** right precentral gyrus, **C** bilateral inferior temporal gyrus, **D** fusiform gyrus. Blue = rs1042615 A allele group, red = rs1042615 GG group.

Furthermore, there were significant differences between the rs1042615 A and GG groups in the right PCG for “sensory sensitivity” (z = –3.3, *p* = 0.001) and “sensation avoiding” (*z* = −0.249, *p* = 0.013) scores. In addition, the genotypic difference between the “low registration” scores in the right FFG was significant (*z* = –3.18, *p* = 0.002). Graphs of correlations between AASP subscales and cortical regions for each genotypic group are presented in Fig. 2.

## Discussion

Accumulating evidence suggests that functional polymorphisms in VTR may contribute to differences in various social behaviors via sensory processing [15–18, 24–33]. Building on the potential role of VT and its receptors in modulating responsiveness to sensory stimuli [17], our study aimed to investigate the relationship between VTR polymorphisms, sensory profiles, and anatomical features using the AASP self-report questionnaire and structural MRI data. Our behavioral results confirmed that the threshold level for sensory stimuli differs depending on the rs1042615 genotype. Specifically, we observed a significant decrease in cortical thicknesses of several brain regions (i.e., right PreCG, PCG, FFG, and bilateral ITG) as the three sensory profile scores (i.e., “low registration”, “sensory sensitivity”, and “sensation avoiding”) increased for A-allele carriers. This suggests a potential link between genetic variations in VTR and alterations in the cortical structure that may underlie changes in sensory processing.

We first found that group A of rs1042615 had two significantly higher scores (i.e., “sensory sensitivity” and “sensation avoiding”) compared to the GG group. This may be because A-allele carriers have a lower sensory threshold and are, therefore, more susceptible to sensory input and experience sensory stimuli more intensely than GG carriers. The scores for “sensory sensitivity” and “sensation avoiding” on the AASP questionnaire are consistent with the theoretical framework for Sensory Processing Sensitivity (SPS) [46]. The SPS framework also accounts for individual differences in the ability to register and process environmental sensory stimuli. However, it is important to note that the SPS primarily focuses on individuals with high sensory processing sensitivity. Furthermore, a recent study has suggested that SPS is likely to be influenced by genetic factors [47]. Various neuropeptides (including oxytocin and corticotropin-releasing hormone) and neurotransmitters (such as GABA, dopamine, serotonin, and norepinephrine) have been proposed as potential candidates involved in SPS [48]. Based on our results, it is likely that genetic polymorphisms in VTR1A, in addition to these candidates, contribute to increased SPS.

High sensitivity in individuals can confer a survival advantage by rendering them more attuned to their surroundings. Simultaneously, however, it is closely linked to negative emotions, while excessive sensitivity is recognized as a diagnostic characteristic in various clinical conditions, including ASD [49, 50]. Moreover, a few studies have also suggested rs1042615 as one of the potential markers associated with ASD [19, 51]; however, the specific impact of rs1042615 on ASD remains unknown. Therefore, we speculated that rs1042615 A carriers may have heightened sensory sensitivity, which is one of the hallmarks of ASD.

Consistent with our behavioral results, the rs1042615 genotype of VTR1A differentially affected the relationship between cortical thickness and sensory profiles. Significant negative correlations were found only for A-allele carriers: 1) between the “low registration” score and the right FFG and PreCG, 2) between the “sensory sensitivity” score and the bilateral ITG, right FFG, PCG, and PreCG, and 3) between the “sensation avoiding” score and the right PCG and PreCG. In other words, the higher the three AASP scores, the thinner the gyri. Notably, there were differences in the correlations between the genotypic groups, with significant group differences observed in the right PCG for the “sensory sensitivity” and “sensation avoiding” scores, as well as in the right FFG for the “low registration” score.

The PCG, a part of the sensorimotor network, is known for its role in integrating complex sensory and motor information [52–54]. It seems that changes in the PCG thickness is affected by genetic factors. Reporting that cortical thinning was most pronounced in sensorimotor and visual cortices, a twin study suggested that the thinning process was highly genetically determined [55]. In comparison with typically developing individuals, individuals with ASD had reduced cortical thickness in the PCG [56, 57]. Given atypical sensory processing in ASD, it suggests the abnormal structural changes in the PCG may alter sensory processing function.

The PreCG also belongs to the primary motor cortex, which is responsible for sensorimotor integration [58]. Cortical thickness in this region exhibited negative correlations with three sensory profiles (i.e., “low registration, “sensory sensitivity,” and “sensory avoiding”) in the A carrier group. A diffusion tensor imaging study reported an inverse correlation between fractional anisotropy and SPS scores in white matter in premotor and somatosensory regions, including PreCG [59]. Structural and functional alterations in the PreCG indicate several clinical issues related to sensorimotor problems [60–63]. For instance, ASD, schizophrenia, and post-traumatic stress disorder, as well as SPS, presented abnormal brain activation pattern in the PreCG in common [50].

In addition, we found that the “low registration” and “sensory sensitivity” scores increased as the right FFG became thinner in the rs1042615 A group. The “low registration” profile is related to a high sensory threshold, whereas the “sensory sensitivity” profile is associated with a low sensory threshold; however, both indicate abnormal sensory characteristics. There is ample evidence that structural or functional changes in the FFG are associated with difficulties in processing sensory information [63–69]. For instance, Green et al. [66] reported a negative correlation between sensory over-responsivity (SOR) and connectivity of the salience network and visual association area, including the FFG, in typically developing individuals as well as in individuals with ASD. Another study found that individuals with ASD who exhibited rightward FFG asymmetry tended to have more severe autistic symptoms [69]. Thus, researchers have suggested that genetic factors may play a role in the association between the structural asymmetry of the FFG and symptom severity in ASD. Additionally, the ITG is also associated with deficits in sensory processing [63, 65]. Abnormalities in sensory and motor functions in the absence of localized brain damage, referred to as neurological soft signs, are associated with reduced cortical thickness in several regions, including the ITG, PreCG, and FFG [63].

The PCG, PreCG, FFG, and ITG, share a common trait of sensory processing and sensorimotor integration, thereby indicating that morphological and functional alternations in these regions may result in sensory processing irregularities. Furthermore, our findings imply that VTR1A polymorphisms play a crucial role in modulating the relationship between sensory processing and cortical thickness in specific brain regions. The sensorimotor and visual cortex have been identified as being strongly influenced by genetics during development [55]. In our study, the rs1042615 GG group showed non-significant but more hypersensitive cortical thickening. Therefore, it may be challenging to discern the relationship between sensory processing and cortical thickness in neurotypical adults without distinguishing between genetic polymorphisms. In other words, genetic polymorphisms not only determine the temperament with which individuals process sensory stimuli but can also provide important information about the neurobiological mechanisms underlying observed reduction in cortical thickness.

What are the possible mechanisms that underlie this interaction? The threshold level of cortical neurons for sensory stimuli fundamentally depends on the E/I balance; disruption of this balance can result in atypical sensory processing, such as hypersensitivity [70, 71]. The GABAergic system, the primary inhibitory neurotransmitter, is responsible for maintaining an E/I balance [72, 73]. Numerous studies have indicated that VT is preferentially involved in GABA actions, and both are implicated in social behavior [32, 74–80]. Specifically, VT can act on VTR1A to differentially modulate GABAergic synaptic transmission [79]. Research has also indicated that VTR1A might potentially impact the development of the E/I balance by altering the connectivity of parvalbumin (PV) GABAergic interneurons, which play a key role in regulating the E/I balance in the neocortex [80]. Furthermore, studies have revealed a link between reduced PV interneurons and various psychiatric disorders, including ASD and schizophrenia [81]. Given the GABAergic and VT system’s roles in social behavior [32, 74–80], this interaction could potentially contribute to individual differences in sensory processing through changes in sensory threshold levels during development.

Another possible mechanism can be inferred from studies on the effects of N-methyl-d- aspartate (NDMA) receptor antagonists on sensory gating. Previous studies in animal models have revealed that exposure to NMDA receptor antagonists induce sensorimotor gating deficits due to GABAergic neuronal dysfunction caused by VTR1A overexpression [82]. A side effects of NMDA receptor antagonists (e.g., ketamine) is the development of schizophrenia-like symptoms, such as hallucinations [83]. These findings indicate that VTR1A expression levels may be associated with alterations in the threshold level of sensory processing; however, these assumptions are speculative, and to the best of our knowledge, no study has investigated how genetic variations in SNP rs1042615 modulate VTR1A expression levels.

Overall, our study underscores the complexity of the genetic and neurobiological factors that shape sensory processing and highlight the need for further investigation. First, future studies investigating these connections through functional neuroimaging are crucial. In line with our findings several functional MRI studies have indicated that highly sensitive individuals exhibit heightened activation in brain regions (e.g., premotor area) responsible for awareness, sensory integration, and preparation for action in response to emotional and social stimuli [84, 85]. This suggests that a sensitive brain mediates the action planning required to respond in social context. Furthermore, the hyperconnectivity of primary sensory regions with the salience network [66] or thalamus [86] could potentially contribute to the severity of over-responsiveness to sensory stimuli. Further our results suggest possibility of genetic interaction with sensory behavior and brain: different genetic polymorphisms might influence this strong activation in the network, consequently resulting in behavioral outcomes. Meanwhile, our finding has promising implications for exploring the underlying mechanisms of sensory-related disorders such as ASD and schizophrenia. Among affected individuals, structural alterations in the brain regions identified in our study are commonly observed [56, 63, 69, 87, 88]. Therefore, expanding on the current study, future investigations should delve into the interplay between the VTR, sensory characteristics, and neurobiological changes in individuals with such conditions.

Although this study sheds light on the potential relationship between VTR1A polymorphisms, sensory processing, and cortical thickness, several limitations should be considered. First, the sample size used in this study was relatively small to divide three genotype group; future studies with larger sample sizes are required to confirm our findings in three genotype group of VTR. Second, the AASP is a self-reported questionnaire that includes social components in items. It is difficult to determine from our findings whether the effect of VTR1A genotype is modulating pure sensory processing excluding social factors or including social factors. Future study is needed to clarify association among VTR, sensory, and brain structure using direct sensory behavior tests (e.g., audiometry, pain, and temperature sensitivity) [52] or the PPI paradigm [7, 9] enables the elucidation of the effect of VTR1A genotype on pure sensory processing without the social components.

Finally, the number of genotypes for SNPs other than rs1042615 was highly disproportionate. This imbalance makes it difficult to characterize how each genotype affects sensory processing and cortical morphology. Therefore, to fully understand the genetic underpinnings of sensory processing and cortical thickness, future studies with more balanced allele distributions are required to comprehensively examine the effects of other SNPs.

### Supplementary information

Supplementary Table

## Data Availability

Deidentified data for this study will be made available upon reasonable request to the corresponding authors.
