# Supplementary material for: Vasotocin receptor gene genotypes moderate the relationship between cortical thickness and sensory processing
Source: Transl Psychiatry. 2023 Nov 21;13:356. doi: 10.1038/s41398-023-02657-2 (PMC10663457; doi:10.1038/s41398-023-02657-2)
Supplement: Supplementary file 1 — Supplementary Table [file 41398_2023_2657_MOESM1_ESM.docx]

**Supplementary table 1. Group differences for SNP genotypes in AASP quadrants**

|  | Sensory Profiles | | | |
| --- | --- | --- | --- | --- |
| SNP | Low Registration | Sensation Seeking | Sensory Sensitivity | Sensation Avoiding |
| rs1042615 | .183 | .787 | .001 | .006 |
| rs3021528 | .885 | .620 | .901 | .906 |
| rs10877969 | .885 | .475 | .958 | .662 |
| rs7268346 | .662 | .418 | .872 | .445 |
| rs28632197 | .123 | .444 | .080 | .339 |
| rs35630000 | .070 | .160 | .249 | .583 |
| rs33911258 | .220 | .132 | .387 | .621 |

**Supplementary table 2. Pearson correlation coefficient (r) and p-value between morphological features of brain and AASP quadrants by SNP rs1042615 groups**

| **AASP** | **Morphological**  **features** | **ROI** | **A** | | **GG** | |
| --- | --- | --- | --- | --- | --- | --- |
|  |  |  | ***r*** | ***p*** | ***r*** | ***p*** |
| Low registration | Thickness | R.FFG | –.421 | .000 | .273 | .160 |
|  |  | R.PreCG | –.351 | .004 | .045 | .822 |
|  |  | R.PCG | –.265 | .033 | –.090 | .656 |
|  |  | R.ITG | –.238 | .056 | –.021 | .918 |
|  |  | L.ITG | –.160 | .204 | –.054 | .791 |
|  | Surface area | R.FFG | .217 | .083 | –.320 | .103 |
|  |  | R.PreCG | .282 | .023 | .064 | .750 |
|  |  | R.PCG | .142 | .258 | .122 | .543 |
|  |  | R.ITG | .281 | .023 | .150 | .456 |
|  |  | L.ITG | .273 | .028 | –.046 | .819 |
|  | Volume | R.FFG | –.013 | .920 | –.234 | .239 |
|  |  | R.PreCG | .100 | .429 | .066 | .744 |
|  |  | R.PCG | .038 | .764 | .013 | .948 |
|  |  | R.ITG | .206 | .099 | .154 | .443 |
|  |  | L.ITG | .236 | .059 | –.059 | .769 |
| Sensation seeking | Thickness | R.FFG | –.023 | .858 | –.092 | .647 |
|  |  | R.PreCG | –.081 | .523 | .034 | .868 |
|  |  | R.PCG | –.014 | .913 | .075 | .709 |
|  |  | R.ITG | .018 | .886 | –.214 | .283 |
|  |  | L.ITG | .124 | .323 | –.276 | .164 |
|  | Surface area | R.FFG | .074 | .556 | –.030 | .882 |
|  |  | R.PreCG | .020 | .873 | .085 | .673 |
|  |  | R.PCG | –.082 | .514 | .062 | .758 |
|  |  | R.ITG | –.029 | .818 | –.286 | .146 |
|  |  | L.ITG | –.056 | .655 | –.076 | .707 |
|  | Volume | R.FFG | .095 | .450 | –.034 | .866 |
|  |  | R.PreCG | –.036 | .775 | .114 | .572 |
|  |  | R.PCG | –.099 | .431 | .075 | .711 |
|  |  | R.ITG | –.049 | .699 | –.367 | .060 |
|  |  | L.ITG | .003 | .983 | –.127 | .528 |
| Sensory sensitivity | Thickness | R.FFG | –.343 | .005 | .088 | .656 |
|  |  | R.PreCG | –.382 | .002 | .047 | .812 |
|  |  | R.PCG | –.400 | .001 | .320 | .097 |
|  |  | R.ITG | –.343 | .005 | –.027 | .892 |
|  |  | L.ITG | –.370 | .002 | –.120 | .544 |
|  | Surface area | R.FFG | .162 | .199 | –.216 | .279 |
|  |  | R.PreCG | .342 | .006 | .239 | .230 |
|  |  | R.PCG | .248 | .046 | .227 | .256 |
|  |  | R.ITG | .267 | .032 | .006 | .978 |
|  |  | L.ITG | .296 | .017 | –.001 | .998 |
|  | Volume | R.FFG | –.003 | .978 | –.195 | .329 |
|  |  | R.PreCG | .160 | .203 | .189 | .344 |
|  |  | R.PCG | .075 | .552 | .302 | .126 |
|  |  | R.ITG | .161 | .200 | –.041 | .841 |
|  |  | L.ITG | .155 | .217 | –.013 | .950 |
| Sensation avoiding | Thickness | R.FFG | –.345 | .006 | .005 | .981 |
|  |  | R.PreCG | –.346 | .004 | .077 | .697 |
|  |  | R.PCG | –.339 | .005 | .212 | .288 |
|  |  | R.ITG | –.279 | .024 | –.121 | .549 |
|  |  | L.ITG | –.165 | .188 | –.201 | .314 |
|  | Surface area | R.FFG | .037 | .771 | –.284 | .151 |
|  |  | R.PreCG | .197 | .115 | .213 | .285 |
|  |  | R.PCG | .162 | .196 | .300 | .128 |
|  |  | R.ITG | .159 | .207 | .259 | .192 |
|  |  | L.ITG | .210 | .093 | .053 | .792 |
|  | Volume | R.FFG | –.090 | .476 | –.306 | .120 |
|  |  | R.PreCG | .010 | .938 | .144 | .475 |
|  |  | R.PCG | .070 | .579 | .310 | .115 |
|  |  | R.ITG | .140 | .266 | .130 | .519 |
|  |  | L.ITG | .195 | .120 | .027 | .892 |
| R = right hemisphere; L = left hemisphere; FFG = right fusiform gyrus; PreCG, = precentral gyrus; PCG = paracentral gyrus; ITG = inferior temporal gyrus | | | | | | |
